# Seroconversion and asymptomatic infections during oseltamivir prophylaxis against Influenza A H1N1 2009

**DOI:** 10.1186/1471-2334-10-164

**Published:** 2010-06-10

**Authors:** Vernon J Lee, Jonathan Yap, Joshua K Tay, Ian Barr, Qiuhan Gao, Hanley J Ho, Boon Huan Tan, Paul M Kelly, Paul A Tambyah, Anne Kelso, Mark I Chen

**Affiliations:** 1 Biodefence Centre, Ministry of Defence, Transit Road, Singapore 778910, Singapore; 2 Centre for Health Services Research, National University of Singapore, Medical Drive, Singapore 117597, Singapore; 3 Department of Epidemiology and Public Health, National University of Singapore, Medical Drive, Singapore 117597, Singapore; 4 National Centre for Epidemiology and Population Health, Australian National University, Canberra, ACT 0200, Australia; 5 WHO Collaborating Center for Reference and Research for Influenza, Wreckyn Street, North Melbourne, Victoria 3051, Australia; 6 Detection and Diagnostics Laboratory, Defence Medical and Environmental Research Institute, DSO National Laboratories, Medical Drive, Singapore 117510, Singapore; 7 Division of Infectious Diseases, National University of Singapore, Lower Kent Ridge Road, Singapore 119074, Singapore; 8 Department of Clinical Epidemiology, Tan Tock Seng Hospital, Jalan Tan Tock Seng, Singapore 308433, Singapore

## Abstract

**Background:**

Anti-viral prophylaxis is used to prevent the transmission of influenza. We studied serological confirmation of 2009 Influenza A (H1N1) infections during oseltamivir prophylaxis and after cessation of prophylaxis.

**Methods:**

Between 22 Jun and 16 Jul 09, we performed a cohort study in 3 outbreaks in the Singapore military where post-exposure oseltamivir ring chemoprophylaxis (75 mg daily for 10 days) was administered. The entire cohort was screened by RT-PCR (with HA gene primers) using nasopharyngeal swabs three times a week. Three blood samples were taken for haemagglutination inhibition testing - at the start of outbreak, 2 weeks after completion of 10 day oseltamivir prophylaxis, and 3 weeks after the pandemic's peak in Singapore. Questionnaires were also administered to collect clinical symptoms.

**Results:**

237 personnel were included for analysis. The overall infection rate of 2009 Influenza A (H1N1) during the three outbreaks was 11.4% (27/237). This included 11 index cases and 16 personnel (7.1%) who developed four-fold or higher rise in antibody titres during oseltamivir prophylaxis. Of these 16 personnel, 8 (3.5%) were symptomatic while the remaining 8 personnel (3.5%) were asymptomatic and tested negative on PCR. Post-cessation of prophylaxis, an additional 23 (12.1%) seroconverted. There was no significant difference in mean fold-rise in GMT between those who seroconverted during and post-prophylaxis (11.3 vs 11.7, p = 0.888). No allergic, neuropsychiatric or other severe side-effects were noted.

**Conclusions:**

Post-exposure oseltamivir prophylaxis reduced the rate of infection during outbreaks, and did not substantially increase subsequent infection rates upon cessation. Asymptomatic infections occur during prophylaxis, which may confer protection against future infection. Post-exposure prophylaxis is effective as a measure in mitigating pandemic influenza outbreaks.

## Background

Anti-viral prophylaxis has been used as a strategy to prevent the transmission and spread of influenza. Post-exposure prophylaxis with oseltamivir, a commonly used neuraminidase-inhibitor, has been shown to be effective in preventing the development of clinical disease against seasonal influenza when used against household contacts [1,2]. Pre-exposure prophylaxis has also been successfully used in the community [3], and in households [4] to prevent transmission of influenza. For the 2009 pandemic, post-exposure prophylaxis has been used in household and community contacts of pandemic influenza cases [5], as well as in pandemic influenza outbreaks in closed environments [6].

One of the uncertainties with prophylaxis is the risk of maintaining an immunologically naïve population which may increase the possibility of outbreaks after the cessation of prophylaxis. One mathematical model showed that premature cessation of prophylaxis before the pandemic's peak resulted in higher peak infection rates compared to no prophylaxis use [7]. However, prophylaxis may delay the spread of the virus such that the overall infection rate in the affected group is reduced, and may spread out the burden of disease, thus reducing the strain on resources and disruption of services. Currently, there is little evidence on the actual outcome of prophylaxis in such situations.

Chemoprophylaxis failures have been previously documented but mostly by the development of clinical influenza illness among individuals receiving prophylaxis [1,4]. However, influenza may also result in asymptomatic infections [8], and one previous study showed that asymptomatic infections while receiving oseltamivir prophylaxis do occur [3]. Asymptomatic sero-conversion may confer protection and increase the overall effectiveness of antiviral prophylaxis in protecting individuals and cohorts even after cessation by increasing herd immunity.

We performed a study in the tropical city-state of Singapore to determine symptomatic and asymptomatic serological confirmation of 2009 Influenza A (H1N1) infections during oseltamivir prophylaxis and after cessation of prophylaxis, in 3 separate outbreaks. The findings will be important in the application of future chemoprophylaxis strategies.

## Methods

We performed an observational cohort study in the Singapore military from 22 Jun 09 to 16 Jul 09. The Singapore military has a mix of regular employees and conscript personnel where all males are required to serve after high school. These personnel live in camps during the week and return home on weekends, resulting in a semi-closed community with exposures to the national community. The Singapore military identified its first imported case of 2009 Influenza A (H1N1) on 15 Jun 2009, and on 22 Jun 2009 identified its first outbreak cluster with local transmission.

In line with national protocols, cases of 2009 Influenza A (H1N1) were determined via laboratory confirmed infection by real-time reverse transcription polymerase chain reaction (RT-PCR) or viral culture [9]. In addition to the national protocol of hospital or home isolation of cases during the early containment phase of the local epidemic [10], the Singapore military used the strategy of geographical oseltamivir ring chemoprophylaxis of affected military units with 10 days of oseltamivir 75 mg once a day, and cohorting of the entire units (as a form of social distancing) to prevent spread.

### Epidemiological Investigations

The study was performed among 252 personnel involved in 3 separate 2009 Influenza A (H1N1) outbreaks, whereby post-exposure oseltamivir ring chemoprophylaxis was administered. At the onset of each outbreak, a 10 day course of post-exposure oseltamivir chemoprophylaxis was given to each cohort and they continued to function in their normal capacity. The entire cohort was screened by RT-PCR using nasopharyngeal swabs three times a week, until no further positive cases were discovered for three days. All confirmed cases were given a minimum of 7 days home medical leave. The rest of the cohort continued their regular schedule, including staying in camp during weekdays and returning home during weekends.

In addition, three samples of 5 to 10 ml of venous blood were taken from each participant in for serological testing. The first baseline sample was taken at the start of outbreak. The second sample was taken between 2 to 3 weeks after the completion of oseltamivir prophylaxis. This timeframe allowed sufficient time for seroconversion from infections during prophylaxis, while reducing the likelihood of seroconversion from infections after prophylaxis [11]. The third sample was taken 3 weeks after the peak of the pandemic in Singapore [12], between 4 to 6 weeks after the completion of prophylaxis, to assess for any additional seroconversion after prophyalxis. Questionnaires were administered to collect data on demographics, medical history, and clinical symptoms.

Written informed consent was obtained from participants, and the study was approved by the Singapore military's Joint Medical Committee (Research) and the Australian National University's ethics review board.

### Laboratory Analysis

The nasopharyngeal swabs collected were resuspended in 3.0 ml of universal transport medium (Copan Diagnostics Inc., USA) and sent for laboratory testing. Total nucleic acid material was extracted using the DNA minikit (Qiagen, Inc, Valencia, CA, USA) according to manufacturer's instructions and subjected to real-time PCR testing for the presence of H1N1-2009 [13]. Briefly, 5ul of nucleic extract was PCR-amplified with 0.8 uM of each of the forward (5'-GAC AAA ATA ACA AAC GAA GCA ACT GG - 3') and reverse primers (5'-GGG AGG CTG TTT ATA GCA CC-3') in the presence of 0.2 uM probe (5'-6-carboxyfluorescein-GCA TTC GCA AT(BHQ)G GAA AGA AAT GCT GG -3') using the Superscript III RT/Platinum Taq mix (Invitrogen Corporation, CA, USA) according to manufacturer's instructions. The reverse transcription (RT) was carried out at 50°C for 30 mins, the reaction denatured at 95°C for 2 mins, and PCR-amplified with 50 cycles consisting of 95°C for 15 sec and 55°C for 30 sec. The RT-PCR testing was carried out on a real-time PCR system (Applied Biosystems 7500, USA), A positive result is defined by a fluorescence growth curve that crosses the threshold line within 40 cycles. Sensitivity of this assay is about 100 copies of RNA genome equivalents per reaction (95% confidence level) [14].

For the blood samples, serum was extracted and tested by haemagglutination inhibition (HI) according to standard protocols (WHO CC, 1982) at the WHO Collaborating Center for Reference and Research for Influenza in Melbourne, Australia. The serum was pretreated with receptor destroying enzyme (RDE) (Deka Seiken Co. Ltd., Tokyo, Japan) at 1:4 (volume/volume), and the enzyme inactivated by addition of an equal volume of 1.6% tri-sodium citrate (Ajax Chemicals, Australia). Egg-grown A/California/7/2009 A(H1N1-2009) virus was purified by sucrose gradient, concentrated and inactivated with β-propiolactone, to create an influenza zonal pool preparation (a gift from CSL, Australia). 25 μL of Influenza Zonal Pool-A/California/7/2009 virus was incubated with an equal volume of RDE-treated serum, titrated in two-fold dilutions in phosphate buffer solution from 1:10 to 1:1280, and incubated for 1 hour. 25 μl 1% (v/v) turkey red blood cells was added to each well and read after 30 minutes. Controls for the HI assay were performed with positive ferret sera (sera collected from naive ferrets infected with A/California/7/2009 H1N1 pandemic virus and bled 14 days later), positive human sera from RT-PCR positive individuals collected in the convalescent phase, and negative human sera collected from non-infected individuals. Positive sera had high titres by both HI and MN assays against pandemic H1N1 viruses. Titres were expressed as the reciprocal of the highest dilution of serum where haemagglutination was prevented. Individual seroconversion was indicated by a four-fold or greater rise in titres between successive samples.

### Statistical Analysis

The data was analyzed using the Statistical Package for the Social Sciences (SPSS, version 16.0, Chicago, IL) with the level of significance set at 0.05. Categorical variables were summarized as percentages and continuous variables as means with standard error (SE); the Student's T-test was used to investigate the relationship between continuous variables.

## Results

**Figure 1:**
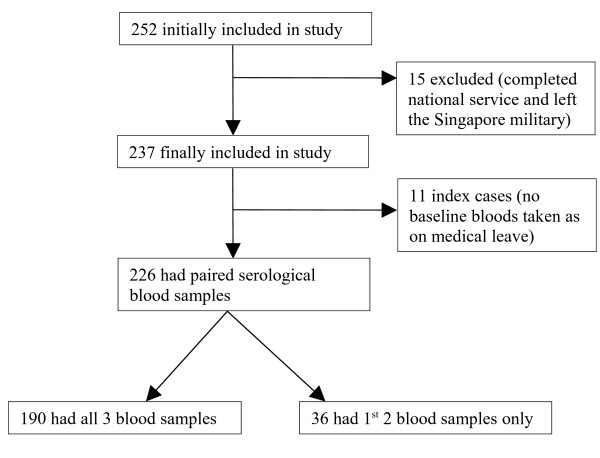
**Enrollment and Follow-up of Study Population**.

Outbreaks A, B and C occurred in 3 separate units on 22 Jun 09, 9 Jul 09 and 16 Jul 09 respectively. Prior to these outbreaks, there were no increases of influenza-like illness or respiratory illness cases in these units, nor were there any confirmed cases of 2009 Influenza A (H1N1). Of the 252 personnel initially identified and sampled; 15 personnel were subsequently excluded as they had completed their conscript service and left the military before completion of the study (Figure 1). The final study population consisted of 237 personnel of which 11 personnel were index cases of the outbreaks. These index cases were started on treatment dose of oseltamivir (75 mg twice daily for 5 days) and given medical leave at the onset of the outbreak and thus did not have any baseline serology taken.

The mean age of the study population was 21.2 years old (range 18.7-30.8) (Table 1) and all were male, reflecting the composition of the military. The ethnic make-up was similar to the general Singapore population. Twenty-three personnel (9.7%) had a history of asthma and 1 (0.4%) each had hypertension and IgA nephropathy; no other relevant medical conditions were present.

**Table 1:** Demographics of study population

	*Overall (n = 237)*	*Outbreak A (n = 149)*	*Outbreak B (n = 42)*	*Outbreak C (n = 46)*
Mean age (SE) (range)	21.2 (1.7) (18.7-30.8)	21.1 (2.1) (18.7-30.8)	21.2 (0.8) (20.2-23.8)	21.2 (0.5) (20.1-22.4)
Median age (yr)				
Male	237 (100%)	149 (100%)	42 (100%)	46 (100%)
Ethnicity				
Chinese	175 (73.8%)	100 (67.1%)	36 (85.7%)	39 (84.8%)
Malay	41 (17.3%)	34 (22.8%)	4 (9.5%)	3 (6.5%)
Indian	12 (5.1%)	8 (5.4%)	1 (2.4%)	3 (6.5%)
Others	9 (3.8%)	7 (4.7%)	1 (2.4%)	1 (2.2%)
Significant medical history	25 (10.5%)*	21 (14.1%)	4 (9.5%)	0 (0.0%)

*All cases of significant medical history were asthma except 1 case of hypertension and 1 case of IgA nephropathy

### Seroconversion During Prophylaxis (Table 2)

**Table 2:** Seroconversion during and post-prophylaxis in the study population

	*Overall* *(n = 237)*	*Outbreak A* *(n = 149)*	*Outbreak B* *(n = 42)*	*Outbreak C* *(n = 46)*
Date of 1^st ^blood sample	23 Jun-16 Jul 10	23 Jun 10	9 Jul 10	16 Jul 10
Date of 2^nd ^blood sample	13 Jul-6 Aug 10	13 Jul 10	30 Jul 10	6 Aug 10
Date of 3^rd ^blood sample	21-25 Aug 10	21 Aug 10	25 Aug 10	25 Aug 10
Seroconversion during prophylaxis (second vs first samples)*				
Total	16/226 (7.1%)	10/141 (7.1%)	4/40 (10%)	2/45 (4.4%)
Symptomatic	8/226 (3.5%)	3/141 (2.1%)	3/40 (7.5%)	2/45 (4.4%)
Asymptomatic (and RT-PCR negative)	8/226 (3.5%)	7/141 (5.0%)	1/40 (2.5%)	0/45 (0.0%)
Overall infection rate during outbreak (serological and index cases)	27/237 (11.4%)	18/149 (12.1%)	6/42 (14.3%)	3/46 (6.5%)
Seroconversion post-prophylaxis (third vs second samples)				
Total	23/190 (12.1%)	16/115 (13.9%)	1/34 (2.9%)	6/41 (14.6%)
Symptomatic	4/190 (2.1%)	2/115 (1.7%)	1/34 (2.9%)	1/41 (2.4%)
Asymptomatic	19/190 (10.0%)	14/115 (12.2%)	0/34 (0.0%)	5/41 (12.2%)

*Excluding index cases

The overall infection rate of 2009 Influenza A (H1N1) during the three outbreaks was 11.4% (27 personnel, including 11 index cases). A total of 16 personnel (excluding index cases) developed a four-fold or higher rise in antibody titres between the first and second blood sample, indicating infection whilst on oseltamivir prophylaxis. Of these, 8 (3.5% of the population) were symptomatic - 6 had fever together with respiratory symptoms while 2 had only respiratory symptoms. The remaining 8 personnel (3.5%) were asymptomatic and tested negative on PCR from consecutive nasopharyngeal swabs.

### Seroconversion Post-Prophyalxis

An additional 23 (12.1%) patients developed 4-fold rise in antibody titres between the second and third blood sample, indicating infection after the cessation of prophylaxis and up to the peak of the epidemic wave. Four (2.1%) were symptomatic.

### Antibody Titres

The baseline and post-seroconversion geometric mean titres (GMT) of those who seroconverted during prophylaxis was 7.4 and 59.1 respectively (Table 3). The baseline and post-seroconversion GMT of those who seroconverted post-prophylaxis was 6.6 and 62.9 respectively. There was no significant difference in mean fold-rise in GMT between the two groups (11.3 vs 11.7, p = 0.888). Ten index cases (ie given treatment dose of oseltamivir) had a single post-seroconversion blood sample taken. The post-seroconversion GMT for these index cases was 65.0 (SE 6.8) compared to 59.1 (SE 6.1) in those who seroconverted during prophylaxis (p = 0.590).

**Table 3:** Comparison of change in antibody titres during and post-prophylaxis

	Baseline GMT(SE)	Post-seroconversion GMT (SE)	Mean fold-rise in titres (SE)	p- value*
Seroconversion during prophylaxis	7.4 (5.8)	59.1 (6.1)	11.3 (2.7)	0.888
Seroconversion post-prophylaxis	6.6 (5.7)	62.9 (5.7)	11.7 (1.5)	

*Comparing mean fold-rise in titres

### Compliance and Side Effects

Of the 237 who started prophylaxis, 228 personnel (96.2%) completed the full course of oseltamivir. Nine personnel (3.8%) did not complete the full course due to non-compliance and side effects; 5 (2.1%) complained of nausea/vomiting. Of these 9 personnel, one was among the symptomatic individuals who seroconverted, while the other 8 did not have any symptoms and did not seroconvert. No allergic, neuropsychiatric or other severe side-effects were noted.

## Discussion

Prophylaxis with oseltamivir has been shown to be effective in reducing the immediate spread of influenza in community and household settings during the period of administration [3,4]. However, the effectiveness of oseltamivir in reducing subsequent infection rates has not been widely studied. Our study showed that prophylaxis may be effective not only in reducing the spread of influenza during a localized outbreak, but also after cessation of prophylaxis during the overall epidemic. In our study, the overall infection rate during the outbreak was 11.4%. This was lower than clinical attack rates in other seasonal influenza outbreaks documented in the military- 57.7% among Taiwanese military recruits [15] and 42% on a navy ship [16]. Our infection rates were also lower when compared to similar 2009 Influenza A (H1N1) outbreaks in other closed communities - >30% attack rate in a school outbreak [17] and 13-17% among household contacts (which consisted of older age groups) [18].

The seroconversion rate after the cessation of prophylaxis until after the community epidemic's peak was 12.1%, which was similar to that of the initial outbreak (11.4%). In addition, the overall combined infection rate throughout the entire epidemic of 21.1% (50/237) was lower than that of other similar military cohorts surveyed in the Singapore military during the same period with a seroconversion rate of 28% [19]. The latter cohorts did not receive early oseltamivir prophylaxis. This shows that anti-viral prophylaxis did not render the population more susceptible to further outbreaks even though prophylaxis was stopped 1-4 weeks before the peak of the epidemic. On the contrary, anti-viral prophylaxis allowed cases to be spread out across time, reducing peak absenteeism and disruptions to the military or business continuity. A previous study in another closed environment, a boarding school, using amantadine post-exposure prophylaxis for seasonal influenza A/H3N2 modeled that prophylaxis reduced the number of clinical influenza-like illness cases during its use by approximately 83.7%, and although the number of cases increased upon cessation of prophylaxis, the overall clinical attack rates were 21.7%, which was lower than predicted using previous outbreaks for comparison [20].

Asymptomatic, RT-PCR negative seroconversion occurred in 3.5% of the participants during oseltamivir prophylaxis. This shows likely exposure to and infection with 2009 Influenza A (H1N1), and the subsequent development of antibodies which may be protective, without increasing transmission. Furthermore, we found that the antibody titres in those who seroconverted during prophylaxis were not significantly different from those who seroconverted after cessation of prophylaxis. As such, in addition to preventing clinical infection, prophylaxis may also result in asymptomatic infection and subsequent immunity which provides individual protection against further infection after cessation of prophylaxis, as well as increasing herd immunity. The identical rates of symptomatic and asymptomatic seroconversions during prophylaxis show that the proportion of asymptomatic infection is substantial and must be considered during any influenza outbreak [21]. Our findings are similar to the another study by Hayden and colleagues, which showed that in the general community during seasonal epidemic influenza, 2.3% to 2.5% of those who received oseltamivir prophylaxis had asymptomatic infection, although this was not significant compared to those on placebo [3].

Our study provides evidence on serological infections and asymptomatic seroconversion while on oseltamivir prophylaxis, incorporating serological, PCR and clinical data. However, there are some limitations of this study. The lack of planned control groups which makes it difficult to assess the likely exposures and infection rates within similar settings, but previous experiences have suggested that exposures and infection rates during the initial outbreak phase are high in closed environments [15-17]. In addition, the age group in these outbreaks is limited to young adults. Additional studies should be performed in different populations and age groups, with comparison groups to determine the overall effectiveness of prophylaxis in reducing clinical infections and promoting immunity.

## Conclusion

Our study showed that post-exposure oseltmaivir prophylaxis reduced the rate of infection in a vulnerable population and did not adversely increase subsequent infection rates upon cessation of prophylaxis before the epidemic's peak. In addition, we have shown that asymptomatic seroconversions occur during prophylaxis, which may confer protection against future infection. Post-exposure prophylaxis remains a strategy to consider in preventing the spread of influenza in closed environments and essential personnel populations.

## Competing interests

VJL has received research support from GSK. PAT has received research support and honoraria from Baxter, Adamas, Merlion Pharma, and Novartis as well as travel support from Pfizer and Wyeth and sits on the boards of the Asia Pacific Advisory Committee on Influenza and the Asian Hygiene Council. The rest of the authors declare that we do not have any conflict of interests, financial or otherwise, in this study.

## Authors' contributions

VJL, JJY conceived the study, collected the data, performed the analysis, and wrote the first draft of the manuscript together. JKT, MIC conceived the study, performed the analysis, and participated in the manuscript writing. GQ, HJH collected the data and performed the analysis. IB, AK, PAK, BHT, PAT performed the analysis and participated in the manuscript writing. All authors have read and approved the final manuscript. The corresponding author had full access to all the data in the study and takes responsibility for the integrity of the data and the accuracy of the data analysis.

## Pre-publication history

The pre-publication history for this paper can be accessed here:

http://www.biomedcentral.com/1471-2334/10/164/prepub

## References

[B1] Welliver R Monto AS Carewicz O Schatteman E Hassman M Hedrick J Jackson HC Huson L Ward P Oxford JS Oseltamivir Post Exposure Prophylaxis Investigator Group: Effectiveness of oseltamivir in preventing influenza in household contacts: a randomized controlled trial JAMA 2001 285 748 54 10.1001/jama.285.6.748 11176912

[B2] Hayden FG Belshe R Villanueva C Lanno R Hughes C Small I Dutkowski R Ward P Carr J Management of influenza in households: a prospective, randomized comparison of oseltamivir treatment with or without postexposure prophylaxis J Infect Dis 2004 189 3 440 9 10.1086/381128 14745701

[B3] Hayden FG Atmar RL Schilling M Johnson C Poretz D Paar D Huson L Ward P Mills RG Use of the selective oral neuraminidase inhibitor oseltamivir to prevent influenza N Engl J Med 1999 341 1336 43 10.1056/NEJM199910283411802 10536125

[B4] Peters PH Jr Gravenstein S Norwood P De Bock V Van Couter A Gibbens M von Planta TA Ward P Long-term use of oseltamivir for the prophylaxis of influenza in a vaccinated frail older population J Am Geriatr Soc 2001 49 1025 31 10.1046/j.1532-5415.2001.49204.x 11555062

[B5] Ministry of Health Singapore 8th Confirmed Case of Influenza A (H1N1-2009) 2009 http://app.crisis.gov.sg/InfluenzaA/Press.aspx?id=41

[B6] Roy A How well are we managing the influenza A/H1N1 pandemic in the UK? BMJ 2009 339 b2897 10.1136/bmj.b2799 19605449

[B7] Lee VJ Chen MI Modeling the Effectiveness of Neuraminidase Inhibitors in Preventing Critical Staff Absenteeism during Pandemic Influenza EID 2007 13 449 57 10.3201/eid1303.060309 PMC2725890 17552099

[B8] Nicholson KG Wood JM Zambon M Influenza Lancet 2003 362 1733 45 10.1016/S0140-6736(03)14854-4 14643124 PMC7112395

[B9] World Health Organisation DG Statement following the Meeting of the Emergency Committee 2009 http://www.who.int/csr/disease/swineflu/4th_meeting_ihr/en/index.html

[B10] Ministry of Health Singapore Case Definitions and Management of Human Infection of Influenza A (H1N1-2009) 2009 https://www.hpp.moh.gov.sg/HPP/contentportlets/hpp/MungoBlobs/380/57/Definition%20(Updated%207%20Jul%2009).pdf

[B11] Miller E Hoschler K Hardelid P Stanford E Andrews N Zambon M Incidence of 2009 pandemic influenza A H1N1 infection in England: a cross-sectional serological study Lancet 2010 375 1100 8 10.1016/S0140-6736(09)62126-7 20096450

[B12] Ministry of Health Singapore Weekly Infectious Disease Bulletin Vol 6 No 41 http://www.moh.gov.sg/mohcorp/uploadedFiles/Statistics/Infectious_Diseases_Bulletin/2009/2009_week_41.pdf 17 Oct 09

[B13] World Health Organisation CDC protocol of realtime RTPCR for influenza A (H1N1) http://www.who.int/csr/resources/publications/swineflu/realtimeptpcr/en/index.html 6 Oct 09

[B14] World Health Organisation WHO information for laboratory diagnosis of pandemic (H1N1) 2009 virus in humans - revised http://www.who.int/csr/resources/publications/swineflu/diagnostic_recommendations/en/index.html 23 Nov 09

[B15] Liu PY Wang LC Lin YH Tsai CA Shi ZY Outbreak of influenza A and B among military recruits: evidence from viral culture and polymerase chain reaction J Microbiol Immunol Infect 2009 42 114 21 19597642

[B16] Earhart KC Beadle C Miller LK Pruss MW Gray GC Ledbetter EK Wallace MR Outbreak of influenza in highly vaccinated crew of U.S. navy ship Emerg Infect Dis 2001 7 463 5 11384530 10.3201/eid0703.010320 PMC2631796

[B17] Lessler J Reich NG Cummings DA New York City Department of Health and Mental Hygiene Swine Influenza Investigation Team Nair HP Jordan HT Thompson N Outbreak of 2009 pandemic influenza A (H1N1) at a New York City School N Engl J Med 2009 361 2628 36 10.1056/NEJMoa0906089 20042754

[B18] Cauchemez S Donnelly CA Reed C Ghani AC Fraser C Kent CK Finelli L Ferguson NM Household transmission of 2009 pandemic influenza A (H1N1) virus in the United States N Engl J Med 2009 361 2619 27 10.1056/NEJMoa0905498 20042753 PMC3840270

[B19] Chen MI Lee VJ Lim WY Barr IG Lin RT Koh GC Yap J Cui L Cook AR Laurie K Tan LW Tan BH Loh J Shaw R Durrant C Chow VT Kelso A Chia KS Leo YS Influenza H1N1 seroconversion rates and risk factors among distinct adult cohorts in Singapore JAMA 2010 303 1383 91 10.1001/jama.2010.404 20388894

[B20] Davies JR Grilli EA Smith AJ Hoskins TW Prophylactic use of amantadine in a boarding school outbreak of influenza A J Royal College of General Practitioners 1988 38 346 8 PMC1711501 3256644

[B21] Monto AS Pichichero ME Blanckenberg SJ Ruuskanen O Cooper C Fleming DM Kerr C Zanamivir prophylaxis: an effective strategy for the prevention of influenza types A and B within households J Infect Dis 2002 186 1582 88 10.1086/345722 12447733

